# A Profibrotic Phenotype in Naïve and in Fibrotic Lung Myofibroblasts Is Governed by Modulations in Thy-1 Expression and Activation

**DOI:** 10.1155/2018/4638437

**Published:** 2018-05-29

**Authors:** Pazit Y. Cohen, Raphael Breuer, Shulamit B. Wallach-Dayan

**Affiliations:** ^1^Lung Cellular and Molecular Biology Laboratory, Institute of Pulmonary Medicine, Hebrew University Hadassah Medical School, 91120 Jerusalem, Israel; ^2^Department of Pathology, Boston University School of Medicine, Boston, MA 02118, USA

## Abstract

Lung fibrosis is characterized by abnormal accumulation of Thy-deficient fibroblasts in the interstitium of the alveolar space. We have previously shown in bleomycin-treated chimeric Thy1-deficient mice with wild-type lymphocytes that Thy1-deficient fibroblasts accumulate and promote fibrosis and an “inflammation-free” environment. Here, we aimed to identify the critical effects of Thy1, or the absence of Thy1, in lung myofibroblast profibrotic functions, particularly proliferation and collagen deposition. Using specific Thy1 siRNA in Thy1-positive cells, Thy1 knockout cells, Thy1 cDNA expression vector in Thy1-deficient cells, and Thy1 cross-linking, we evaluated cell proliferation (assessed by cell mass and BrdU uptake), differentiation (using immunofluorescence), and collagen deposition (using Sircol assay). We found that myofibroblast Thy1 cross-linking and genetic manipulation modulate cell proliferation and expression of Fgf (fibroblast growth factor) and Angtl (angiotensin) receptors (using qPCR) that are involved in myofibroblast proliferation, differentiation, and collagen deposition. In conclusion, lung myofibroblast downregulation of Thy1 expression is critical to increase proliferation, differentiation, and collagen deposition.

## 1. Introduction

Pulmonary fibrosis is characterized by abnormal accumulation of myofibroblasts in the interstitium and alveolar space [1, 2]. Persistent myofibroblast survival and accumulation are the essential events underlying the evolution of lung fibrosis in animal models and idiopathic pulmonary fibrosis (IPF) in humans [3].

Several mechanisms have been suggested to drive the unabated multiplication of myofibroblasts in IPF [4], including a high proliferation rate [5, 6] and resistance to apoptosis [7–9], based on evidence from in vitro studies using myofibroblasts from humans with IPF, as well as in vivo research using murine models of lung fibrosis.

Fibroblasts are heterogeneous, differing in phenotype and function [10, 11]. In the lungs of mice and humans, there are two subpopulations of fibroblasts, which are distinguished by their expression of Thy1 [10, 12]. It has been shown that fibroblasts in the lungs of humans with IPF and of bleomycin-treated mice are predominantly Thy1^−^ [13–16].

Thy1 is a 25–37 kDa glycosylphosphatidylinositol- (GPI-) anchored cell surface protein that belongs to the immunoglobulin-like gene superfamily [17]. Thy1 has a variety of functions in different tissues [18], including a role in cell apoptosis [19–24] and proliferation [16, 25–31]. It has been reported to function in T cell activation, neurite outgrowth, apoptosis, tumor suppression, and wound healing and fibrosis [32] via multiple pathways. It is involved in T cell activation, and its role in T cell function has been extensively reviewed [33–35]. In mice and in humans, Thy1^+^ and Thy1^−^ fibroblasts differ with respect to cytokine [28, 36–39] and growth factor responses [40, 41], as well as cell migration [42].

Using gene chip analysis, we found that myofibroblast Thy1 cross-linking mediates downregulation of genes promoting cell proliferation, survival, and differentiation and reduces production of extracellular matrix (ECM) components, while concurrently mediating the upregulation of genes known to foster inflammation and immunological functions [15].

In the current study, we further evaluated the critical role of Thy1 protein in lung myofibroblast proliferation and apoptosis in naïve cells and during the evolution of lung fibrosis, as well as its association with profibrotic functions such as differentiation and collagen production.

## 2. Materials and Methods

### 2.1. Animals

**Figure 1:**
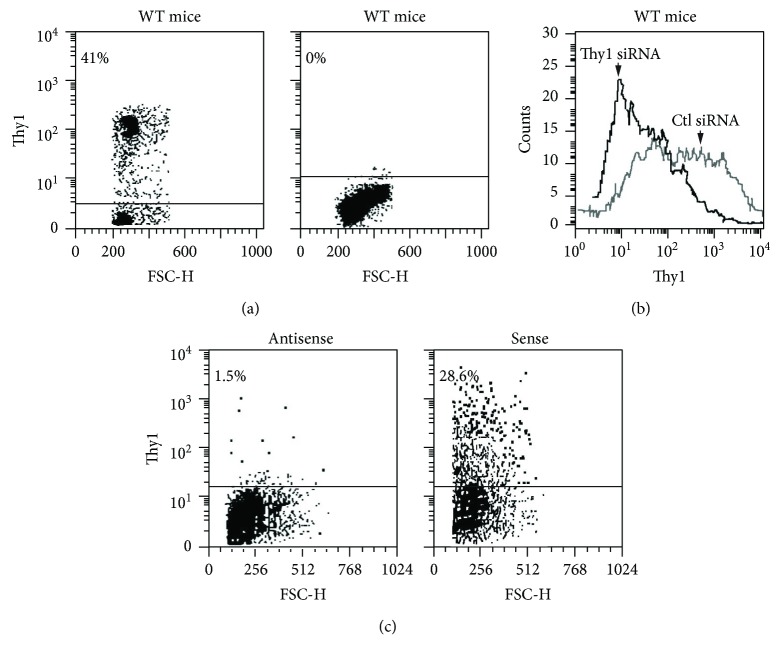
Thy1 gene expression following genetic manipulation, using Thy1 knockout mice, Thy1 siRNA, and Thy1 cDNA expression vector. (a) Flow cytometry Thy-FITC mAb staining in spleen cells from wild-type (WT) C57BL/6 mice and Thy1-deficient mice, respectively. (b) Downregulation of Thy1 by siRNA was performed. Thy1 downregulation was detected 48 h after transfection by FACS analysis (c) Mlg cells were transfected with Thy1 expression vector. Plasmid-containing Thy1 cDNA in antisense orientation serves as a control. Thy1 expression was detected 24 h after the transfection by FACS analysis.

Male, 11-12-week-old, C57BL/6J mice (Jackson Laboratory, Bar Harbor, ME, USA) and C57BL/6J Thy1-deficient mice were used (kindly provided by Professor R.J. Morris, Department of Chemistry, King's College, London, UK). Their Thy1-deficient status versus wild-type (WT) was confirmed in C57BL/6 Thy1-deficient mice by flow cytometry of spleen cells using anti-Thy1-FITC mAb (Figure 1(a)). At 12–14 weeks, body weight for the two types was similar, in the range of 23–27 gm. Histological sections of the lung, heart, brain, colon, liver, and kidney were studied. There were no differences in the histological sections or in lung hydroxyproline content, which were 1274 ± 181 (mean ± SE) and 1122 ± 73 in Thy1-deficient and WT controls, respectively. Bronchoalveolar cellularity was also similar in terms of the number of cells per ml, with 99% macrophages and 1% lymphocytes or neutrophils. The two groups of mice were thus similar in all respects except for Thy1 expression.

All animal procedures were approved by the Hebrew University Hadassah Medical School Animal Care Committee. Mice were housed in a specific pathogen-free environment.

### 2.2. Intratracheal Instillation

Mice were anesthetized by intraperitoneal (IP) injection of 0.05–0.07 ml of 40 mg/ml Ketalar (Parke-Davis, Pontypool, Gwent, UK) and 0.5 mg/ml droperidol (Janssen Pharmaceutica, Beerse, Belgium). A dose of 0.06–0.08 units of bleomycin (H. Lundbeck, Copenhagen, Denmark) dissolved in 0.1 ml of saline solution to induce lung fibrosis, or 0.1 ml of saline alone as control, was slowly injected intratracheally (IT). The mice were sacrificed 14 days after IT, as we have previously described and detailed [3].

### 2.3. Lung Cell Isolation and Culture and Quantification of Myofibroblasts

The lungs were removed, minced, and incubated (37°C, 5% CO_2_ air) for 45 min in PBS containing 1 mg/ml collagenase (C0130, Sigma-Aldrich, St. Louis, MO, USA). After enzyme treatment, lung tissue was gently passed through a cell dissociation sieve (Sigma-Aldrich) or 40 *μ*m nylon mesh filters (Falcon, Becton Dickinson, Franklin Lakes, NJ) and then washed twice in PBS. For myofibroblast culture experiments, lung cells (LC) were resuspended in fibroblast culture medium. Cell cultures were incubated at 37°C in 5% humidified CO_2_. Typically, within 1 week of culture initiation, more than 95% of the cells were morphologically myofibroblasts.

Cells were passaged every 5 days by dissociating monolayers with a mild trypsin solution (Biological Industries, Beit HaEmek, Israel). After initial cultures were established, myofibroblasts obtained on passages 2 through 20 were used. Myofibroblast *α*-SMA cell markers were evaluated by assessment of any increase in cell staining visualized on confocal microscope and by quantification of mean fluorescence intensity (MFI) with flow cytometry using specific anti-*α-*SMA mAb (Dako, Glostrup, Denmark) diluted 1 : 200 in 1% BSA.

### 2.4. Thy1^+^ and Thy1^−^ Myofibroblast Subpopulation Sorting by Flow Cytometry

Myofibroblasts (0.5 × 10^6^) were collected from cultures and incubated in FACS buffer (3% FCS in PBS) with anti-Thy1.2 PE (Pharmingen, San Diego, CA, USA) (0.06 *μ*g per 0.5 × 10^6^ cells) for 30 min at room temperature, washed with FACS buffer, and analyzed by flow cytometry or sorted using a FACS cell sorter under sterile conditions using a FACS-Star™ (Beckton-Dickinson, Franklin Lakes, NJ, USA), as shown in (Figure 1(a)). Thy1^+^ cells are more spindle-shaped and have more elongated processes than Thy1^−^ cells, which are more rounded.

### 2.5. Proliferation

#### 2.5.1. Cell Cycle Analysis by Flow Cytometry

BrdU (Sigma-Aldrich) was added to the fibroblast culture to reach a final concentration of 20 *μ*M. The cells were later removed by incubation with trypsin for 2 h (for Mlg cells) or 6 h (for primary fibroblasts) and centrifuged at 1200 rpm for 10 min. The pellets were washed in 5 ml of 1× cold PBS, gently resuspended in 100 *μ*l of cold PBS, and fixed by slowly dripping 5 ml of ice-cold ethanol (70%). Cells were incubated at −20°C for at least 30 min or overnight, pelleted by centrifugation at 1800 rpm for 5 min, and 1 ml of 2 N HCl/Triton X-100 was slowly added with a gentle vortex. Cells were then incubated at room temperature for 30 min, pelleted by centrifugation at 1800 rpm for 5 min, and resuspended in 1 ml of 0.1 M Na_2_B_4_O_7_, pH 8.5 to neutralize the sample. After centrifugation (1800 rpm, 5 min), the pellet was incubated with 20 *μ*l FITC-conjugated anti-BrdU (Becton Dickinson) for 30 min at room temperature. Cells were pelleted and resuspended in 1 ml of PBS containing 5 *μ*g/ml propidium iodide (PI) and stored in the dark until analyzed by flow cytometry. The proliferation distribution was determined by measuring corresponding BrdU uptake versus total DNA content (cells in S phase).

#### 2.5.2. Proliferation Rate Analysis by Confocal Microscope

Myofibroblasts isolated from the mouse lungs were seeded (0.1 × 10^6^) on 22 × 22 mm glass coverslips in fibroblast culture medium. After 24 h, BrdU was added to the culture to a final concentration of 20 *μ*M. Nonadherent cells were removed 6 h later, and adherent cells were washed twice with PBS and fixed by incubation with cold 70% ethanol overnight at 20°C. Cells were then incubated with 0.5 ml of 2 N HCl/Triton X-100 (room temperature, 30 min), and 0.5 ml of 0.1 M Na_2_B_4_O_7_, pH 8.5 was added. Cells were then washed with PBS and incubated with 20 *μ*l anti-BrdU-FITC (Becton Dickinson) for 30 min at room temperature. PI 5 *μ*g/ml was added for 2 min. Cells were then washed twice with PBS, and coverslips were mounted on glass microscope slides with mounting solution and examined with a confocal microscope (Carl Zeiss AG, Oberkochen, Germany) attached to a Zeiss Axiovert 135M inverted microscope.

#### 2.5.3. Cell Growth Assessment by Methylene Blue Staining

The growth of myofibroblast monolayers was assessed by colorimetric quantitation of the cell mass of the surviving monolayer after staining with the basic dye methylene blue [43]. Assays were initiated with 10^4^ cells/well in 96-well microtiters, plated, and incubated overnight to confluence at 37°C in a 5% CO_2_ environment. Cells were fixed in 2.5% glutaraldehyde in 200 *μ*l of medium. Fixed monolayers were washed twice with 200 *μ*l of borate buffer (10 mM, pH 8.4) and stained for 1 h with methylene blue (1% in 10 mM borate buffer). Excess stain was removed by three washes with double-distilled water (DDW), and plates were dried overnight at room temperature. Bound methylene blue was extracted with 200 *μ*l of 0.1 M HCl followed by 1 h incubation at 37°C and measured at an optical density of 620 nm in a microtiter plate reader (Titertek Multiskan MMC, Flow Laboratories, Irvine, UK).

#### 2.5.4. Cell Proliferation Assessment by CFSE Staining

In order to track myofibroblast growth, the cells were stained by fluorescent carboxyfluorescein diacetate succinimidyl ester (CFSE) [44] to allow flow cytometry visualization of eight to 10 discrete generations of cell division both in vitro and in vivo. CFSE labeling is distributed equally between daughter cells after division. Daughter cells thus show half of the fluorescence of their parent cells.

2 × 10^6^ myofibroblasts/ml were resuspended in PBS. An equal volume of freshly diluted 2.5 *μ*M CFSE (Molecular Probes) in PBS was added for 8 min at room temperature and staining was stopped by the addition of an 1 ml ice-cold FCS for 1 min. Cells were immediately washed three times in RPMI 1640, and the cells were cultured for 72 h. The intensity of CFSE labeling was measured by flow cytometry with CFSE intensity labeling at time 0 serving as a control measurement.

### 2.6. Myofibroblast Thy1 Activation

Subconfluent myofibroblasts were stimulated with anti-Thy1 G7, which has previously been shown to activate T cells [45], or with anti-rat IgG2C*κ* isotype control (Pharmingen). We followed the methods discussed previously by Cohen et al. [46]. Both stimulants were added to myofibroblasts at varying concentrations ranging from 1–20 *μ*g/ml, together with recombinant protein G cross-linker (Sigma-Aldrich) at the same concentration.

### 2.7. RNA

#### 2.7.1. RNA Isolation

Total cellular RNA was isolated from myofibroblasts in culture using TRI Reagent (cat. number T9424, Sigma-Aldrich) according to the protocol supplied by the manufacturer. To assess RNA integrity and exclude DNA contamination, an aliquot of each sample was analyzed by electrophoresis on a 1% agarose stained with ethidium bromide. Purity and quantitation of RNA was assessed by spectrophotometer.

#### 2.7.2. Reverse Transcription Polymerase Chain Reaction (RT-PCR)

RNA was reverse transcribed to cDNA using an avian myeloblastosis virus RT-based protocol and random primers, as well as poly-dT (Reverse Transcription System, Promega, Madison, WI, USA). One microgram of each sample was uniformly used for reverse transcription. The cDNA was diluted in a final volume of 200 *μ*l with nuclease-free water.

TaqMan real-time PCR, primers, and probes were purchased from Applied Biosystems (Foster City, CA, USA). Probe sequences of the genes analyzed were as follows:

(i) 18s 5′-ATTGGAGGGCAAGTCTGGTGCCAGC-3′

(ii) FGFR 5′-GCTCGGCACGAGACAGACTGGTCTTA-3′

(iii) AGRT1 5′-TTTCGCCAAGCCTGCACCTCCATGC-3′

The real-time PCR reaction mixture contained 9 or 4.5 *μ*l of the sample and 10 or 5 *μ*l of 2× TaqMan Universal PCR Master Mix (Applied Biosystems), 1 or 0.5 *μ*l of 20× mix of unlabeled PCR primers, and a fluorogenic probe (5′ FAM dye labeled). A PRISM 7000 Sequence Detection System (Applied Biosystems) was used with the default thermal cycling program (95°C for 10 min followed by 40 cycles of 95°C, 20 seconds, 60°C, and 1 minute). Reactions were performed in triplicate. The relative quantification method was used with ΔCt calculated as Ct (target gene)-Ct (18s gene). The relative quantity of the product was expressed according to the formula 2^−ΔCt^.

### 2.8. Construction of Thy1 Expression Vector

Thy1 expression vector was obtained by ligation of the entire Thy1 cDNA into pTARGET™ mammalian expression vector (Promega). Thy1 cDNA was generated from primary culture of mouse lung fibroblasts by RT-PCR, using two specific primers assigned from the published above in the paragraph discussing RT-PCR.

(i)Forward- 5′GACAAGCTTATGAACCCAGCCAT3′

(ii)Reverse- 5′GCCTCTAGATCACAGAGAAATGAA3′

The PCR product of the expected size, coded for the full-length cDNA of Thy1, was purified using Wizard SV Gel and PCR Clean-Up System (Promega, Madison, WI, USA) according to the manufacturer's instructions. Purified products were cloned into a pTARGET mammalian expression vector (Promega), according to manufacturer's instructions. The correctness of the insert was confirmed by sequencing (Danyel Biotech, Mira Korner, Hebrew University, Jerusalem, Israel).

### 2.9. Gene Expression Manipulation

#### 2.9.1. Downregulation of Gene Expression by siRNA

SiRNAs were purchased from Qiagen (Valencia, CA, USA). Nontargeting siRNA (cat. number SI1027281) was used as a control.

#### 2.9.2. Thy1 Downregulation

A combination of two types of Thy1 siRNA, 10 picomole of each (cat. numbers SI01448132 and SI01448125), was used.

#### 2.9.3. Transfection Using the DreamFect™ Kit

1 *μ*g of expression vector was delivered into cultured fibroblasts (0.25 × 106) in each well of a 6-well plate, in the presence of 4 *μ*l/1 *μ*g DNA DreamFect (OZ Biosciences, Marseille, France), to 2 ml final medium volume. Following incubation at 37°C in humidified 95% air, 5% CO_2_ atmosphere, the medium was renewed.

#### 2.9.4. Transfection Using Electroporation

Myofibroblasts were harvested and washed twice with ice-cold phosphate-buffered saline (PBS) (Mg^2+^, Ca^2+^ free) and resuspended in solution R (transfection kit). Small interference RNA (siRNA) or cDNA expression vector (2 *μ*g) was added to 12 *μ*l of cell suspension containing 0.5 × 106 cells. The mixture was then subjected to a single pulse from a micoporator apparatus (Digital Bio Technology, Seoul, South Korea). After shocking, samples were added to growth medium with 10% FCS. Protein expression analysis was performed 24–48 h after transfection.

#### 2.9.5. Exogenic Thy1 Gene Expression Manipulation Using Thy1 siRNA or Thy1 cDNA Expression Vector

Transfected fibroblasts were tested to detect changes in Thy1 expression by staining with anti-Thy1 antibody and flow cytometry analysis. Thy1 downregulation was detected when siRNA specific to Thy1 was introduced into primary myofibroblasts isolated from the mouse lungs, with mean fluorescence intensity (MFI) of Thy1 staining decreasing from 553 to 83 (Figure 1(b)). Thy1 upregulation was detected when Thy1 expression vector was introduced into Mlg cell line (mouse lung-transformed fibroblasts) that lacks Thy1 expression. Expression of Thy1 was accomplished in 28.6% of non-Thy1 expressor Mlg cell line (mouse lung fibroblast), using sense but not antisense/control orientation of Thy1 cDNA (Figure 1(c)).

### 2.10. Statistical Analysis

The Mann–Whitney nonparametric test was performed for comparison of two groups. *p* < 0.05 was considered statistically significant.

## 3. Results and Discussion

### 3.1. Thy1 Expression and Myofibroblast Proliferation

**Figure 2:**
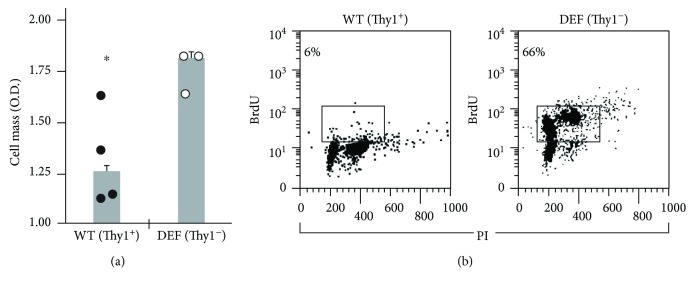
Proliferation of myofibroblasts from WT- and Thy1-deficient (DEF) mice. (a) Cell mass of myofibroblasts isolated from WT and Thy1 (DEF) mice measured after 24 h by methylene blue staining, followed by colorimetric assay. The results are presented as fold change from day 0 (*n* = 3 − 4 in each group) (^∗^*p* < 0.05). (b) BrdU uptake in myofibroblasts from WT (Thy1^+^) and Thy1 (DEF) mice (Thy1^−^) previously treated with 10 *μ*M BrdU for 24 h. BrdU uptake was assessed by anti-BrdU-FITC-conjugated mAb staining versus PI-total DNA staining followed by flow cytometry.

Thy1 expression is associated either with a low or a high rate of cell growth in different cell types [16, 25, 26, 28–31]. Since we found an increase in the proportion of Thy1^−^ myofibroblasts among the total myofibroblast population at day 14 following bleomycin IT [15], we assessed whether the high rate of lung myofibroblast proliferation is associated with an absence of Thy1 expression. To this end, myofibroblasts were isolated from the lungs of Thy1-deficient and WT-untreated mice. Results show a high proliferation rate as detected by cell mass measurement in myofibroblasts isolated from Thy1-deficient mice compared to myofibroblasts isolated from WT mice (Figure 2(a)). These results were confirmed by cell cycle analysis and BrdU uptake showing higher percentage of proliferation in myofibroblasts isolated from Thy1-deficient mice (66%) compared to myofibroblasts isolated from WT mice (6%) (Figure 2(b)).

### 3.2. Inverse Correlation between Thy1 Expression and Fibroblast Proliferation

**Figure 3:**
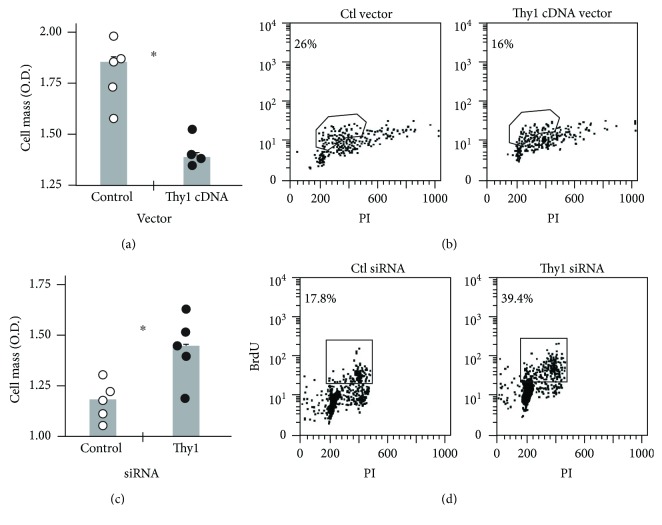
Inverse correlation between Thy1 expression and fibroblast proliferation. (a) 24 h following transfection, cell mass was measured by colorimetric assay using methylene blue staining in Mlg cells transfected with Thy1 expression vector and in control plasmid-containing Thy1 cDNA in antisense orientation. Results are presented as fold change from day 0 (*n* = 4 − 5 in each group) (^∗^*p* < 0.05). (b) BrdU uptake in Mlg-transfected cells with Thy1 expression vector or control plasmid-containing Thy1 cDNA in antisense orientation following treatment with 20 *μ*M BrdU for 2 h. BrdU uptake was assessed by anti-BrdU-FITC-conjugated mAb staining versus PI-total DNA staining followed by flow cytometry analysis. (c) In primary lung myofibroblasts transfected with nontargeting siRNA (Ctl) or Thy1 siRNA, cell mass was measured by colorimetric assay after methylene blue staining, 24 h posttransfection. Results are presented as fold change from day 0. In each group, *n* = 5, ^∗^*p* < 0.05. (d) BrdU uptake in primary lung myofibroblasts transfected with nontargeting siRNA (Ctl) or Thy1 siRNA, following transfection and treatment with 20 *μ*M BrdU for 6 h. BrdU uptake was assessed by flow cytometry analysis, following anti-BrdU-FITC-conjugated mAb staining versus PI-total DNA staining.

We then assessed whether the variance in proliferation rates between Thy1^+^ and Thy1^−^ myofibroblasts can be attributed to differences in Thy1 expression. A Thy1-negative lung fibroblast cell line, Mlg, was transfected with a Thy1.2 expression vector or a control with antisense orientation. Proliferation was assessed following transfection, by cell mass measurement and BrdU uptake. As shown, introduction of Thy1 cDNA expression vector into the Mlg cell line decreased its proliferation rate as measured by cell mass, from 1.8 (O.D.) in the control vector-transfected cells to 1.35 in Thy1-upregulated Mlg cells (Figure 3(a)). These results were confirmed by cell cycle analysis, which showed a decrease in the proliferation rate of Thy1-upregulated Mlg cells to 16% compared to 26% for control Thy1^−^ cells (Figure 3(b)).

Concomitantly, by knocking down Thy1 expression in murine primary lung myofibroblasts (Thy1^+^) using specific siRNA, we demonstrated that the myofibroblast proliferation rates were increased, as assessed by cell mass measurements, from 1.1 to 1.5 (O.D.) in Thy1-downregulated myofibroblasts (Figure 3(c)). These results were further confirmed by cell cycle analysis with BrdU uptake showing concomitant increases in proliferation rates for myofibroblasts with downregulated Thy1 (39.4%) compared to control (17.8%) (Figure 3(d)).

### 3.3. Thy1 Activation Downregulates Expression of Several Genes That Have a Role in Lung Fibroblast Proliferation

**Figure 4:**
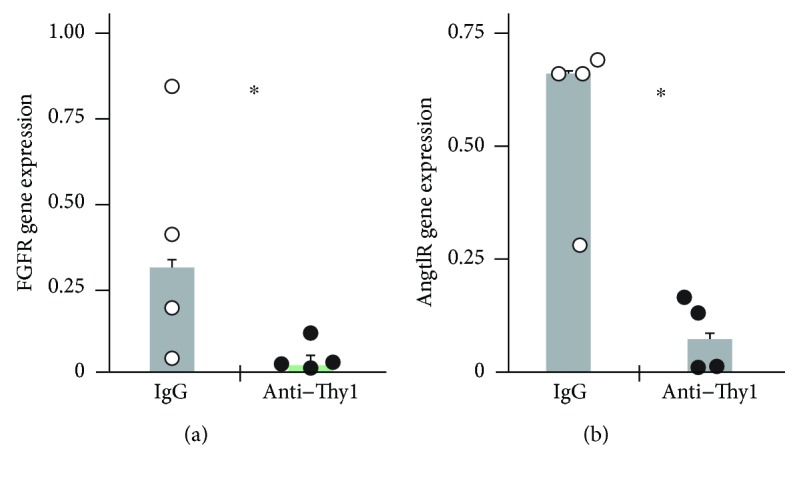
FGFR and AGRT1 mRNA expressions are decreased following Thy1 activation. Primary fibroblasts stimulated with G7 anti-Thy1 mAb (10 *μ*g/ml) or control IgG isotype for 30 min (for FGFR) or 1 h (for AGRT1). Gene expression was detected by real-time RT-PCR (^∗^*p* < 0.05). (a) Relative expression of FGFR mRNA normalized to 18 s. (b) Relative expression of AGRT1 mRNA normalized to 18 s.

As we have previously shown [15], gene chip analysis revealed that Thy1 activation downregulated the expression of several genes that have a role in lung fibroblast proliferation [46], including genes affecting the cell cycle, as well as genes involved in signaling in the MAPK [47], insulin [48, 49], and TGF*β* [50, 51] pathways. In this study, we validated these gene chip results by real-time RT-PCR. We chose to validate fibroblast growth factor receptor (FGFR1) [52] and angiotensin receptor (AGRT1) [53], which appear to have important roles in fibroblast proliferation. Results obtained by real-time RT-PCR were consistent with gene chip analysis, which showed decreases in expression of both genes (Figures 4(a) and 4(b), resp.). This downregulation may provide some explanation for our observation that Thy1 expression attenuates lung myofibroblast proliferation due to the decrease in myofibroblast receptors that transmit proproliferation signals.

### 3.4. Thy1^−^ Myofibroblasts from the Fibrotic Lungs Are More Proliferative Than Thy1^+^ Myofibroblasts

**Figure 5:**
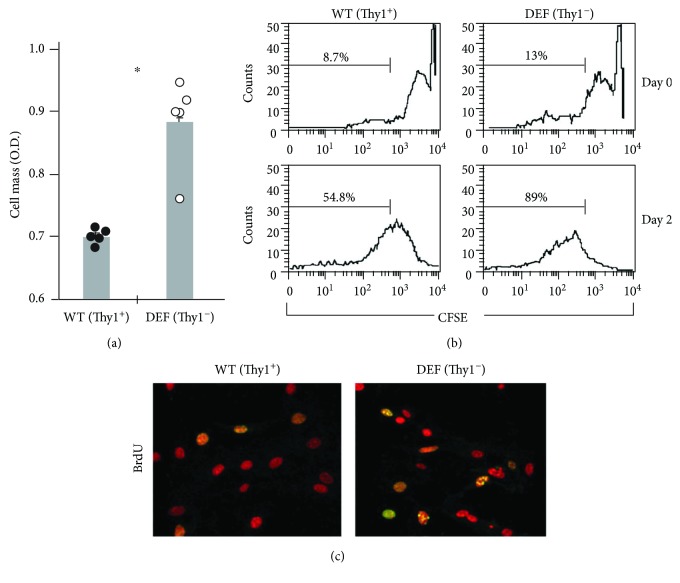
Proliferation rates of Thy1^+^ and Thy1^−^ myofibroblasts isolated from the fibrotic lungs. Lung cells were removed from bleomycin-treated mice at 14-day post-IT. Primary lung myofibroblast cultures were obtained and maintained in RPMI medium. The cultures were sorted by FACS using PE-conjugated anti-Thy1 (CD90) mAb to isolate Thy1^−^ and Thy1^+^ myofibroblasts. (a) Cell mass of Thy1^+^ and Thy1^−^ myofibroblasts measured after 24 h, by methylene blue staining followed by colorimetric assay. Results are presented as fold change from day 0 (^∗^*p* < 0.05). (b) CFSE staining primary cultures of Thy1^+^ and Thy1^−^ myofibroblasts were stained with CFSE. After 72 h of incubation, intensity of CFSE labeling was measured by flow cytometry. The percentage of the cells that lost CFSE labeling at days 0 and 3 is noted. (c) BrdU uptake Thy1^+^ and Thy1^−^ myofibroblasts were treated with 20 *μ*M BrdU for 6 h. BrdU uptake was assessed by anti-BrdU-FITC-conjugated mAb staining and subjected to confocal microscopy analysis.

In order to extend the in vitro findings performed in naïve cells (Figures 1–4) to the in vivo murine model of lung fibrosis, we compared the proliferation of Thy1^**+**^ and Thy1^−^ myofibroblasts isolated from the fibrotic lungs. Myofibroblasts were isolated from the lungs of bleomycin-treated mice and sorted into Thy1^+^ and Thy1^−^ by FACS cell sorter. As shown in Figure 5, the proliferation rate increased from 0.68 to 0.9 OD when assessed by cell mass measurement (Figure 5(a)) and from 54.8% to 89% based on CFSE staining (Figure 5(b)). The BrdU uptake rate (Figure 5(c), yellow spots) was higher for Thy1^−^ myofibroblasts compared with Thy1^+^ myofibroblasts.

### 3.5. Thy1 Expression Is Not Involved in the Regulation of Myofibroblast Apoptosis

In addition to increased proliferation, myofibroblast accumulation in lung fibrosis can also result from their decreased capability to undergo apoptosis. We initially assessed whether Thy1 has a role in determining basal levels of lung fibroblast apoptosis. To this end, cleavage of pro-caspase 3 was assessed in Thy1^+^ and Thy1^−^ fibroblasts taken from the lungs of bleomycin-treated mice.

**Figure 6:**
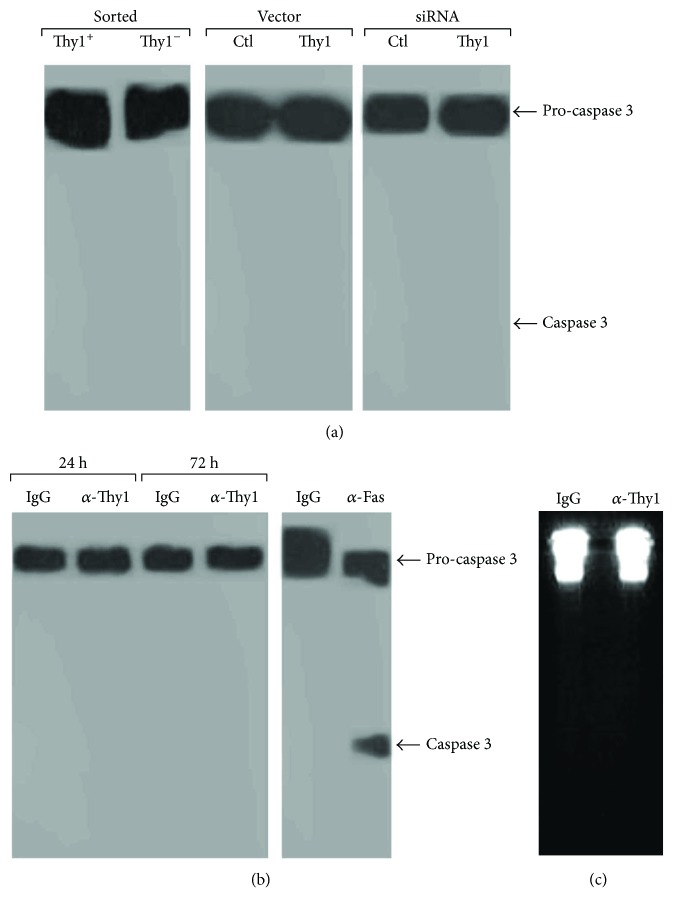
Thy1 expression is not involved in the regulation of fibroblast apoptosis. (a) Western blots determining the extent of pro-caspase 3 cleavage (apoptosis) in Thy1^+^ or Thy1^−^ sorted fibroblasts from the lungs of bleomycin-treated mice and in a lung fibroblast cell line (Mlg) transfected with a Thy1.2 expression vector or plasmid-containing Thy1 cDNA in antisense orientation and in murine primary lung myofibroblasts transfected with Thy1 siRNA or control siRNA. (b) Western blot of pro-caspase 3 cleavage. (c) DNA ladder in lung fibroblast primary cultures following stimulation with G7 anti-Thy1 mAb (5 *μ*g/ml) or IgG isotype match control for 24–72 h.

Cleavage was also assessed in a lung fibroblast cell line (Mlg), following transfection with a Thy1.2 expression vector or a control vector, and in murine primary lung myofibroblasts transfected with Thy1 siRNA or control siRNA. Repeat experiments showed no cleavage of the pro-caspase 3 in any of the tested fibroblasts (Figure 6(a)), indicating that Thy1 expression does not have a role in the regulation of basal fibroblast apoptosis.

In order to assess the requirement of Thy1 activation, as opposed to just Thy1 expression in the regulation of fibroblast apoptosis, we followed the methods described in Cohen et al. [46]. Briefly, primary lung myofibroblasts were stimulated with G7 anti-Thy1 mAb (10 *μ*g/ml), which we have previously shown to induce Src phosphorylation [46]. Cleavage of pro-caspase 3 and DNA ladders was determined in primary fibroblasts following Thy1 activation. No pro-caspase 3 cleavage or DNA ladders were observed following Thy1 activation (Figures 6(b) and 6(c), resp.), further confirming that Thy1 does not influence apoptosis.

### 3.6. Induction of Myofibroblast Differentiation Is Associated with Downregulation of Thy1 Expression

**Figure 7:**
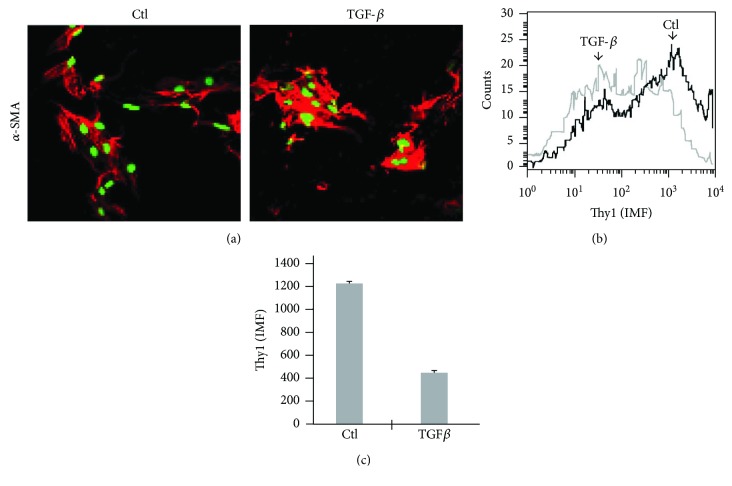
Decrease in Thy1 fibroblast expression during myofibroblast differentiation. Lung myofibroblasts in primary culture were stimulated for 72 h with TGF*β* (2 ng/ml). (a) Cells were fixed and stained with anti-*α-*SMA antibody (red) and PI (green) and analyzed by confocal microscope. (b) Cells were stained using PE-conjugated anti-Thy1 and analyzed by FACS. (c) Graphical presentation of results from (b).

Myofibroblasts are activated fibroblasts expressing *α*-smooth muscle actin protein (*α*-SMA), and are the major cellular component in IPF fibroblastic foci [54]. The origin of these myofibroblasts is controversial. Some studies suggest that they derive from preexisting peribronchial and perivascular adventitial fibroblasts [55] while others suggest that they result from fibroblasts that have been induced to differentiate into myofibroblasts by treatment with cytokines, such as TGF*β* [56, 57], which is known to be secreted [39] and activated [41] following exposure to fibrotic stimuli. It has been shown that Thy1^−^ myofibroblasts have higher *α*-SMA expression compared to their Thy1^+.^counterparts [58]. As previously shown by others [59], and as we hypothesized and show here (Figure 7), during differentiation of fibroblasts to myofibroblasts (new synthesis of *α*-SMA), Thy1 expression is decreased, rendering activated Thy1^−^ myofibroblasts. To this end, murine primary lung myofibroblasts were stimulated with TGF*β* for 72 h and stained for Thy1 and *α*-SMA. While myofibroblast *α*-SMA expression was increased following TGF*β* stimulation, as determined by visual increase in cell staining in confocal microscope (Figure 7(a)) and by quantification of mean fluorescence intensity (MFI) by flow cytometry (Figures 7(b) and 7(c)), Thy1 expression was decreased (Figure 7), indicating that differentiation of fibroblasts into myofibroblasts is associated with downregulation of Thy1 protein expression. This may serve as another mechanism increasing the Thy1^−^ myofibroblast population during lung fibrosis.

In addition, as we have shown in our previous publication [15], gene chip analysis revealed that Thy1 activation as detailed by Cohen et al. [46] downregulates expression of some genes that have a role in myofibroblast differentiation and actin cytoskeleton regulation. Three genes are of special interest here. Two of them, Pax7 [60] and MyoD [61], are master muscle differentiation regulatory genes; the third is *α*-SMA, which is known as a myofibroblast cell marker. The inhibitory effects of Thy1 activation on myofibroblast differentiation and gene expression explain the necessity of Thy1 downregulation to allow myofibroblast differentiation.

### 3.7. Thy1^−^ Myofibroblasts from the Fibrotic Lungs Increase Collagen Protein and RNA Levels Than Thy1^+^ Myofibroblasts

**Figure 8:**
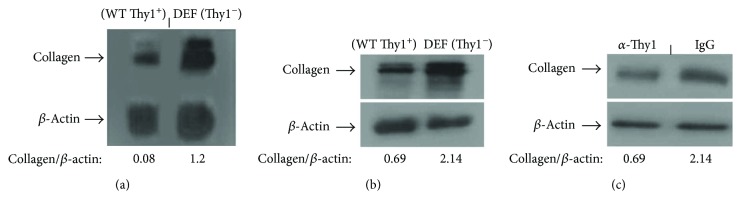
Thy1 expression is involved in the regulation of collagen in fibroblasts. QPCR (a) and Western blots (b-c) determining the extent of collagen expression in Thy1^+^ (a) or Thy1^−^ (b) sorted fibroblasts from the lungs of bleomycin-treated mice and in (c) naïve lung fibroblast primary cultures following stimulation with G7 anti-Thy1 mAb (5 *μ*g/ml) or IgG isotype match control for 24–72 h.

To extend the in vitro findings performed in naïve cells (Figures 1–4) to the in vivo model of lung fibrosis, we compared the collagen protein and gene expression of Thy1^**+**^ and Thy1^−^ myofibroblasts isolated from the fibrotic lungs and collagen protein expression in myofibroblasts that were stimulated with G7 anti-Thy1 mAb (10 *μ*g/ml). As shown in Figure 8, collagen protein expression is downregulated at the RNA level (Figure 8(a)) and protein level (Figure 8(b)) in Thy1^+^ compared to Thy1^−^ fibrotic lung myofibroblasts. Moreover, direct activation of Thy1 receptor by specific anti-Thy1 mAb (*α*-Thy1) versus control (IgG) decreased the extent of spontaneous collagen production in naïve lung myofibroblasts (Figure 8(c)).

Abnormal myofibroblast accumulation and collagen deposition are characteristics of lung fibrosis [62, 63]. Myofibroblasts are thought to be primarily responsible for increased deposition of collagen within the lung [55, 64]. We have previously shown that Thy1 activation downregulates genes promoting fibroblast cell survival and differentiation [15], functions that are considered to be profibrotic. In this case, Thy1 may also serve as a protector against fibroblast overactivity, as shown by others who report an inverse correlation between Thy1 expression on fibroblasts and the evolution of lung fibrosis [16, 65].

The increased number of myofibroblasts accumulating in lung fibrosis may be due to their increased proliferation, their resistance to apoptosis, or both. We have found that Thy1^−^ myofibroblasts isolated from bleomycin-treated mice are more proliferative than Thy1^+^ subsets (Figures 2–5). Moreover, Thy1 downregulation increased myofibroblast proliferation, and exogenic upregulation of Thy1 decreased it (Figure 3), indicating that Thy1 has an inhibitory effect on myofibroblast proliferation.

These observations are consistent with previous reports showing in vitro that Thy1^−^ fibroblasts are more proliferative than those which are Thy1^+^ when exposed to fibrogenic cytokines and growth factors [28], both in lung sections of bleomycin-treated mice and in humans with IPF [16]. Nevertheless, in naïve mice, no significant difference in cell growth rates between Thy1^+^ and Thy1^−^ fibroblasts was detected [11], possibly due to differences in the in vivo milieu of these naïve and the bleomycin-treated mice above.

The inhibitory effect of Thy1 on cell proliferation was also demonstrated in neurite outgrowth [66] and tumor growth [25, 26, 29]. Inhibition of cell proliferation is related to a GPI-linked protein, as demonstrated in T cells [34], and there is precedent for a role of Src kinase in T cell growth inhibition [67]. Consistent with these observations, we have shown that Thy1 activation in myofibroblasts induces Src phosphorylation [46], which may have inhibitory effect on myofibroblast growth.

Alternatively, Thy1 may attenuate lung myofibroblast proliferation indirectly by downregulating receptors such as Fgf and Angtl, which control fibroblast proliferation [52, 53], as we have shown by gene chip analysis [15] and validated by real-time PCR (Figure 4). The decrease in these receptors' expression may reduce transmission of proproliferation signals to fibroblasts and thereby attenuate myofibroblast proliferation.

We found no difference between WT and chimeric Thy1-deficient mice with WT lymphocytes and Thy1-deficient mesenchymal cells, when compared to control the saline-treated mouse lungs in assessments of lung fibrotic injury by semiquantitative morphological index of pathological sections, as well as collagen content [15]. In Thy1 null mice, Thy1 is absent from all cells that would otherwise express it, in contrast to our chimeric Thy1-deficient mice, whose lymphocytes express Thy1. Because Thy1 is normally expressed on murine lymphocytes, it is possible that the severe lung fibrosis following bleomycin IT that develops in Thy1 null mice, is not due to changes in fibroblast Thy1 expression, but rather is due to changes in lymphocyte function [68]. However, the role of lymphocytes in bleomycin-induced lung fibrosis remains controversial, as does the role of inflammation in IPF [4, 69–74].

Gene chip analysis revealed that Thy1 activation downregulated expression of several other genes that have a role in lung fibroblast proliferation, including cell cycle, MAPK [47], and insulin signaling pathway genes [15, 49, 75]. Taken together with our earlier findings [15], these results provide a possible explanation for the mechanism for Thy1 expression attenuation of lung myofibroblast proliferation.

Neither activation nor exogenic upregulation or downregulation of Thy1 expression led to any change in fibroblast apoptosis (Figure 6); however, it may be possible that Thy1 can indirectly influence myofibroblast susceptibility to apoptosis that is triggered by other factors. It has been shown that angiotensin II and TGF-*β* protect fibroblasts from apoptosis [76, 77]. Indeed, we show that Thy1 activation downregulates angiotensin II and pathway genes affecting TGF-*β* signaling [15] and therefore may increase myofibroblast capacity to undergo apoptosis. This is consistent with a previous report showing that Thy1^+^ fibroblasts were more sensitive to apoptosis induction [58]. Myofibroblasts are the major cellular component in IPF fibroblastic foci [54]. Gene chip analysis revealed that Thy1 activation downregulates *α*-SMA expression [15]. This is consistent with a previous report showing that Thy1^−^ myofibroblasts have higher *α*-SMA expression compared to Thy1^+^ [58]. Moreover, Thy1 activation downregulated the expression of genes known to have a role in myofibroblast differentiation, such as Pax7 [60] and MyoD [61], which are master regulatory genes of muscle differentiation. This is consistent with a previous report showing enhancement of myofibroblast differentiation marker in Thy1^−^ compared to Thy1^+^ fibroblasts [58]. Moreover, the inhibitory effect of Thy1 activation on the expression of genes involved in myofibroblast differentiation explains the necessity of Thy1 downregulation for myofibroblast differentiation. Indeed, we show that induction of myofibroblast differentiation (e.g., *α*-SMA expression) by TGF-*β* stimulation was associated with reduction of Thy1 expression (Figure 7(a)). In addition, in bleomycin-treated mice, the number of *α*-SMA-positive cells increased [78, 79] when myofibroblast expression of Thy1 decreased (Figure 7(b)).

In addition, TGF-*β* is a key mediator of lung fibrosis [80, 81]. It stimulates fibroblast proliferation and migration [51], induces ECM production [82, 83], and promotes myofibroblast differentiation [84]. We show here that Thy1-deficient cells overexpress collagen at the RNA (Figure 8(a)) and protein (Figure 8(b)) levels. Moreover, Thy1 stimulation decreased collagen protein levels in naïve lung fibroblasts (Figure 8(c)). Our gene chip analysis revealed that Thy1 activation downregulates downstream molecules of the TGF-*β* signaling pathway [15]. This finding is consistent with other observations showing that Thy1 limits the ability of fibroblasts to activate TGF-*β* [41], and Thy1 null mice have higher TGF-*β* compared to WT mice following bleomycin instillation [16]. Since TGF-*β* stimulates fibroblast proliferation [51], deceased expression of genes involved in TGF-*β* signaling may reduce the transmission of proproliferation signals to fibroblasts, leading to the attenuation of their proliferation.

## 4. Conclusions

These findings indicate that decreases of Thy1^+^ lung myofibroblast subsets in lung fibrosis increase their proliferative functions. Thy1 is critical and not only associated with the downregulation/control of genes promoting cell survival and proliferation, fibroblast differentiation, and collagen production as thoroughly assessed by genetic manipulations in Thy^+^ and in mirror experiments with Thy^−^ lung fibroblasts.

## Abbreviations

AGRT: Angiotensin receptor
BAL: Bronchoalveolar lavage
BrdU: Bromodeoxyuridine staining
cDNA: Complementary DNA
CFSE: Carboxyfluorescein diacetate succinimidyl ester
Ct: Target gene
ECM: Extracellular matrix
FCS: Fluorescence-activated cell sorting
FGFR: Fibroblast growth factor receptor
FITC: Fluorescein isothiocyanate
GPI: Glycosylphosphatidylinositol
h: Hour
IP: Intraperitoneal injection
IPF: Idiopathic pulmonary fibrosis
IT: Intratracheal injection
mAb: Monoclonal antibody b
min: Minutes
PBS: Phosphate-buffered saline
PE: Phycoerythrin
qPCR: Quantitative polymerase chain reaction
RPM: Revolutions per minute
RT: Room temperature
RT-PCR: Reverse transcription polymerase chain reaction
sec: Seconds
siRNA: Small-interfering RNA
TGF: Transforming growth factor
Thy1^−^: Thy1-negative
Thy1^+^: Thy1-positive
WT: Wild-type
*α*-SMA: *α*-Smooth muscle actin.
